# Temporal interface in dispersive hyperbolic media

**DOI:** 10.1515/nanoph-2025-0065

**Published:** 2025-08-05

**Authors:** Grigorii Ptitcyn, Diego M. Solís, Mohammad Sajjad Mirmoosa, Nader Engheta

**Affiliations:** Department of Electrical and Systems Engineering, 6572 University of Pennsylvania, Philadelphia, PA 19104, USA; Departamento de Tecnología de los Computadores y de las Comunicaciones, Universidad de Extremadura, 10003 Cáceres, Spain; Departamento de Ingeniería Audiovisual y Comunicaciones, Universidad Politécnica de Madrid, 28040 Madrid, Spain; Department of Physics and Mathematics, University of Eastern Finland, P.O. Box 111, FI-80101 Joensuu, Finland

**Keywords:** temporal interface, hyperbolic medium, time-modulation

## Abstract

Spatial inhomogeneity, temporal modulation, and engineered anisotropy of parameters of electromagnetic media offer numerous opportunities for manipulating light–matter interaction over the past decades. Here, we investigate a scenario in which we deal with the temporal interface, hyperbolic anisotropy in the form of layered structures, and frequency dispersion. We theoretically investigate how a monochromatic uniform plane wave – propagating in an unbounded, homogeneous, isotropic dielectric medium – undergoes changes due to the rapid temporal variation of such medium into a hyperbolic dispersive medium formed by the stack of thin metal–dielectric bilayers, in which the metal follows the lossless Drude dispersion and the dielectric is assumed to be dispersionless. We corroborate our analytical results by numerical simulations. We observe several interesting phenomena, such as conversion of the original frequency into three pairs of frequencies, resulting in three sets of forward (FW) and backward (BW) waves. We present the amplitudes and the time-averaged Poynting vectors for such FW and BW waves and discuss some of the salient features of such temporal interface.

## 1 Introduction

Electromagnetic wave interaction with time-varying media has recently gained considerable attention and growing interest [1], [2], [3]. This topic offers the notion of four-dimensional (4D) metamaterials [4], wherein material parameters such as relative permittivity can undergo rapid changes in time, either independently or in conjunction with spatial variations. Interest in spatiotemporal modulation, mostly as applied to circuits, has roots dating back to the 1950s [5], [6], [7], [8], [9], yet it has recently garnered significant attention in various research groups worldwide. This newfound interest is mainly due to its promising potential for unconventional wave manipulation and a diverse range of potential applications [1], [2], [4], [10], [11], [12], [13], [14], [15], [16], [17], [18], [19], [20], [21] that include artificial magnetic field for photons [22], optically induced negative refraction [23], frequency conversion [24], amplification [25], [26], Doppler shift [27], [28], Fresnel drag [29], camouflage [30], [31], and nonreciprocity [32], [33], [34], [35], [36], [37] to name a few.

A simple, while fundamental scenario one may devise is a temporal interface. In analogy with a spatial interface between two different semi-infinite media, a temporal interface is defined when a spatially unbounded medium in which a wave propagates is abruptly transformed in time into another medium with different material parameters, e.g., when the relative permittivity of a medium is sharply altered in time [7]. Unlike conventional spatial interfaces, temporal interfaces exhibit three distinctive properties: immutability of momentum (wave vector) accompanied by a frequency shift, lack of electromagnetic energy conservation, and generation of a backward wave, which, due to causality, propagates in the medium after the temporal interface [7], [38], [39]. These features have been experimentally validated, for example, in plasma physics [40], [41], with water waves [42], and in transmission lines that operate at megahertz frequencies [43]. Also, according to these fundamental characteristics and considering complex electromagnetic systems, such as anisotropic and bianisotropic media [44] and metasurfaces [45], a myriad of possibilities and opportunities for manipulation of classical and quantum fields have been uncovered. These advancements encompass: the creation of “wiggler mode” [46], [47], [48], temporal aiming [12], direction-dependent wave manipulation [49], inverse prism [50], antireflection temporal coatings [51], [52], [53], polarization engineering [49], [54], [55] and polarization-dependent analog computing [56], wave freezing and thawing [57], [58], the transformation of surface waves into free-space radiation [57], [59], photon-pair generation [60], [61], angular-dependent inhibition of photon production [62], photon-pair destruction, and vacuum state generation [61].

Despite the growing body of research in this area, the sudden creation of hyperbolic media, resulting in a temporal interface, remains largely unexplored. Here, we extend the notion of temporal interface to dispersive hyperbolic media. Specifically, we explore how a monochromatic electromagnetic uniform plane wave undergoes changes when a host medium, assumed to be a simple isotropic dispersionless dielectric (e.g., free space), is rapidly transformed into a dispersive hyperbolic medium, formed by a stack of many bilayers made of metal and dielectric layers. The frequency dispersion of the hyperbolic medium is taken into account by considering the Drude dispersion for those metal layers. In particular, we reveal that a temporal interface in the presence of such anisotropy and frequency dispersion causes the splitting of the initial frequency of the wave into three pairs, which propagate mainly along the optical axes of the crystal, exhibiting canalization.

The paper is organized as follows. In Section 2, we discuss the mechanism of the corresponding temporal interface. In Sections 3 and 4, we explain the frequency conversion and the evolution of electromagnetic fields as the result of the temporal interface. In Section 5, we demonstrate numerical simulation results, and, finally, in Section 6, we conclude the work.

## 2 Description of the problem

To start, let us consider our hyperbolic medium as an infinitely extended collection of identical bilayers, each formed by a dielectric layer of thickness *d*_d_ and relative permittivity *ϵ*_d_ and a metallic layer of thickness *d*_*m*_ and relative permittivity *ϵ*_*m*_. We assumed all these bilayers are parallel with the *xz* − plane of a Cartesian coordinate system, with its *y* axis being normal to these bilayers. According to the effective medium theory, the elements of the relative permittivity tensor of such a medium can be written [63] as

(1a) $$\epsilon_{xx}=\epsilon_{zz}=f\epsilon_{m}+\left(1-f\right)\epsilon_{\text{d}},$$

(1b) $$\epsilon_{yy}=\frac{1}{\left(1-f\right)/\epsilon_{\text{d}}+f/\epsilon_{m}},$$

with *f* = *d*_*m*_/(*d*_d_ + *d*_*m*_). Let us assume the relative permittivity of the dielectric layers not frequency dispersive, and the relative permittivity of metallic layers lossless Drude-dispersive,

(2) $$\epsilon_{m}=\epsilon_{\infty }-\frac{\omega_{\text{p}}^{2}}{\omega^{2}},$$

where *ω*_p_ is the plasma frequency and *ϵ*_∞_ is the relative permittivity at infinite frequency. Plugging Eqs. (2) into (1a) and (1b) and assuming *ϵ*_d_ = *ϵ*_∞_, we can get frequency-dependent expressions for *ϵ*_*xx*_, *ϵ*_*yy*_, and *ϵ*_*zz*_,

(3a) $$\epsilon_{xx}=\epsilon_{zz}=\epsilon_{\infty }-\frac{\omega_{\text{p,eff}}^{2}}{\omega^{2}},$$

(3b) $$\epsilon_{yy}=\epsilon_{\infty }-\frac{\omega_{\text{p,eff}}^{2}}{\omega^{2}-\omega_{0,yy}^{2}},$$

where

(4a) $$\omega_{\text{p,eff}}\equiv \omega_{\text{p}}\sqrt{f},$$

(4b) $$\omega_{0,yy}\equiv \omega_{\text{p}}\sqrt{\frac{1-f}{\epsilon_{\infty }}}.$$

Dispersion in Eq. (3a) is of the Drude type, whereas dispersion in Eq. (3b) is of the Lorentzian type, with resonance frequency *ω*_0,*yy*_ (Eq. (4b)). Interestingly, and as expected, one can engineer effective media parameters by properly selecting the relative thicknesses of the layers.

**Figure 1:**
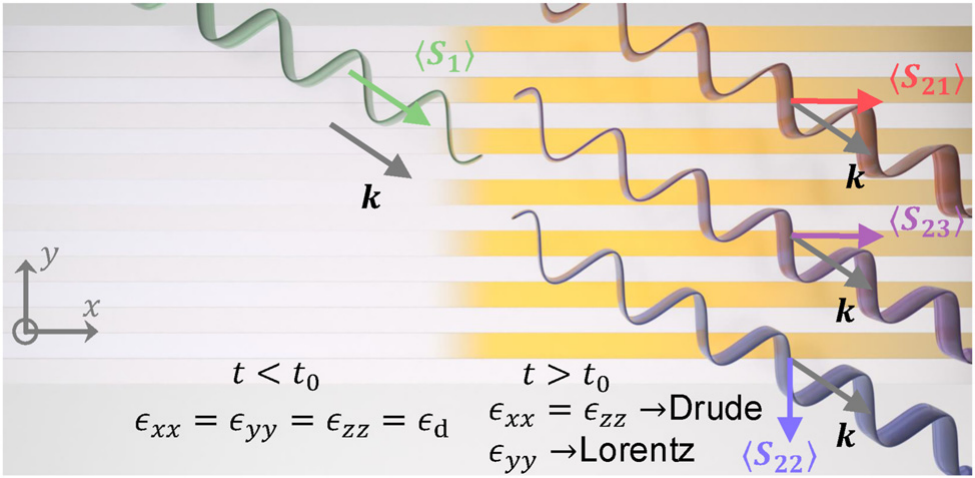
Temporal interface between a simple isotopic medium and a rapidly generated dispersive anisotropic medium: different colors of the waves indicate different frequencies after temporal jump. ⟨*S*_1_⟩, ⟨*S*_21_⟩, ⟨*S*_22_⟩, ⟨*S*_23_⟩ denote the time-averaged Poynting vectors of the corresponding FW waves. We do also have time-averaged Poynting vector for the BW waves (not shown here for the sake of brevity).

In the equations above, as mentioned, it is assumed that *ϵ*_∞_ = *ϵ*_d_. This choice ensures that, when the plasma frequency is zero, we have an isotropic nondispersive effective medium *ϵ*_d_ (see Eqs. (3) and (4) when *ω*_p_ = 0). Clearly, as shown, introducing a nonzero plasma frequency (*ω*_p_ ≠ 0) imparts dispersive and anisotropic properties to the effective medium. Thus, a sudden change in plasma frequency presents a unique opportunity to establish a temporal interface between two markedly distinct media. This is illustrated by Figure 1.

## 3 Frequency conversion due to temporal interface

For simplicity, let us initially consider a monochromatic uniform plane wave with angular frequency *ω*_1_ propagating inside a medium described by Eq. (2) but initially parameterized with a zero plasma frequency, which makes the medium a simple isotropic and dispersionless dielectric. At *t* = *t*_0_, we abruptly ionize the metallic layers by increasing *ω*_p_ from zero to, say, 2*ω*_1_, making the medium both anisotropic and frequency dispersive. Frequency 2*ω*_1_ is chosen to show a large enough contrast of the effects, and, in principle, it can be chosen arbitrarily. The dispersion relation for stationary (i.e., time-invariant) anisotropic media with the electric field components in the *x* − *y* plane and the magnetic field parallel with the *z* axis reads

(5) $$\frac{k_{x}^{2}}{\epsilon_{yy}}+\frac{k_{y}^{2}}{\epsilon_{xx}}=\frac{\omega^{2}}{c^{2}},$$

where the wave vector components *k*_*x*_ and *k*_*y*_ should be conserved quantities across the temporal boundary at *t* = *t*_0_. In the absence of material dispersion, this conservation of wave vectors allows us to find, from Eq. (5), the new converted frequency, *ω*_2_, after *t*_0_ [12]. In the case of frequency dispersive isotropic media, one obtains dispersion relations from a transcendental equation, as discussed in [64]. In the present work, however, we have a combination of anisotropy and frequency dispersion, requiring the medium to be characterized by frequency-dependent *ϵ*_*xx*_ and *ϵ*_*yy*_, leading to another transcendental equation, which reduces to

(6) $$k_{x}^{2}\frac{\omega_{2}^{2}-\omega_{0,yy}^{2}}{\epsilon_{\infty }\left(\omega_{2}^{2}-\omega_{0,yy}^{2}\right)-\omega_{\text{p,eff}}^{2}}+k_{y}^{2}\frac{\omega_{2}^{2}}{\epsilon_{\infty }\omega_{2}^{2}-\omega_{\text{p,eff}}^{2}}=\frac{\omega_{2}^{2}}{c^{2}}.$$

Due to anisotropy, the converted frequencies after *t*_0_, *ω*_2_, depend (through *k*_*x*_ and *k*_*y*_) on the initial incidence angle of propagation *ψ*. Equation (6) can, therefore, be recast as the following 6th-order characteristic equation

(7) $$\begin{array}{ll} & \omega_{2}^{2}\left(\epsilon_{\infty }\omega_{2}^{2}-\omega_{\text{p,eff}}^{2}\right)\left[\epsilon_{\infty }\left(\omega_{2}^{2}-\omega_{0,yy}^{2}\right)-\omega_{\text{p,eff}}^{2}\right]\\ & \;-k_{x}^{2}c^{2}\left(\omega_{2}^{2}-\omega_{0,yy}^{2}\right)\left(\epsilon_{\infty }\omega_{2}^{2}-\omega_{\text{p,eff}}^{2}\right)\\ & \;-k_{y}^{2}c^{2}\omega_{2}^{2}\left[\epsilon_{\infty }\left(\omega_{2}^{2}-\omega_{0,yy}^{2}\right)-\omega_{\text{p,eff}}^{2}\right]=0. \end{array}$$

In our lossless scenario, this expression can be made 3rd order by considering $\omega_{2}^{2}$ as the new variable, with each of the three solution pairs ±*ω*_2_ indicating a forward- and a backward-propagating wave, which hereafter will be denoted as FW and BW waves, respectively.

**Figure 2:**
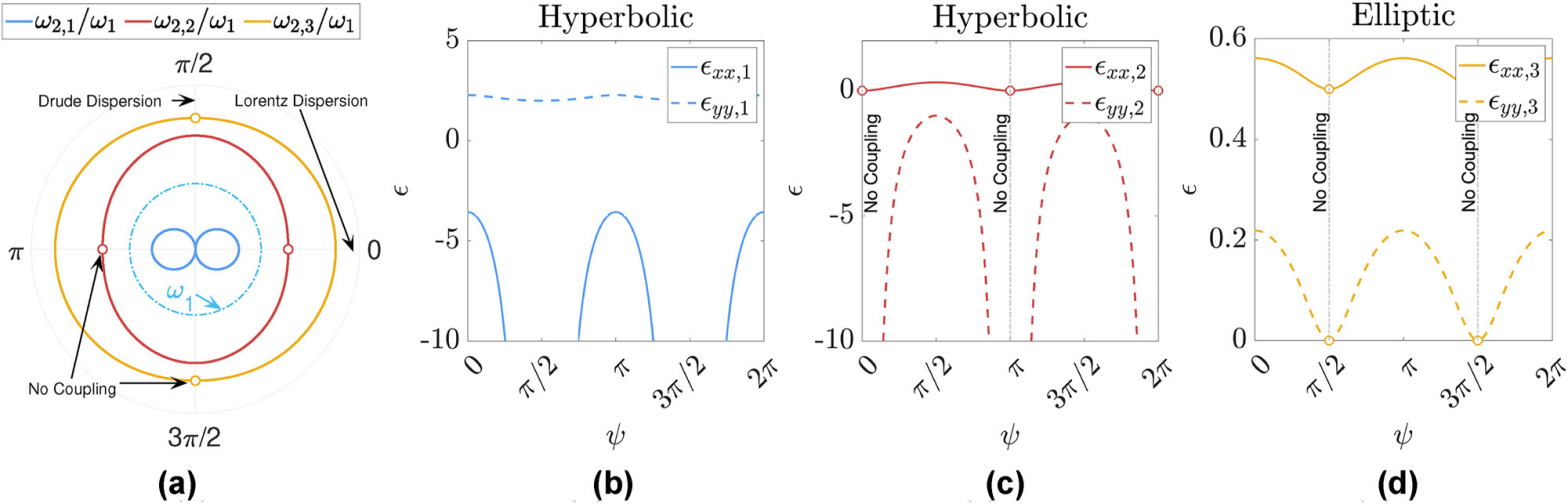
Converted frequencies and relative permittivity values after temporal interface: (a) polar plots of the normalized converted frequencies versus incidence angle, after sudden temporal transition from isotropic medium to anisotropic hyperbolic medium, assuming *d*_d_ = *d*_*m*_, *ω*_p_ = 2*ω*_1_, and *ϵ*_∞_ = 1. (b) and (c) Effective relative permittivity in *x* and *y* directions for different converted frequencies in (a): (b) hyperbolic medium *ω*_2,1_, (c) hyperbolic medium *ω*_2,2_, (d) elliptic medium at *ω*_2,3_. Note that the circles represent vanishing coupling to the corresponding mode.

It is interesting to note that a temporal interface in an unbounded dispersionless media (either isotropic or anisotropic) only shifts the incident frequency, resulting in a single pair of FW and BW waves with a single converted frequency (see, e.g., [7], [12]). It has also been shown that in lossless isotropic medium with Lorentz dispersion, one obtains two positive solution pairs [64], providing two pairs of FW and BW waves with two converted frequencies. In our specific scenario here, which involves anisotropy and frequency dispersion, we observe the emergence of three solution pairs. Figure 2(a) shows the polar plots of converted frequencies after the temporal jump as a function of the direction of propagation angle of the initial wave *ψ*. Propagation along the axes is equivalent to propagation in a medium with the corresponding Lorentz or Drude dispersion. Specifically, propagation along *x* axis (*ψ* = 0 and *ψ* = *π*) or *y* axis (*ψ* = *π*/2 and *ψ* = 3*π*/2) is equivalent to propagation in a medium with permittivity *ϵ*_*yy*_ (see Eq. (3b)) or *ϵ*_*xx*_ (see Eq. (3a)), respectively. Figure 2(a) shows three frequencies for all *ψ* except of *ψ* = *mπ*/2 with *m* is arbitrary integer number, where the amplitude of one of the three pairs of FW and BW waves is zero. More will be said below.

Figure 2(b)–(d) shows the *xx* and *yy* elements of relative permittivity tensors of the medium after the temporal interface, as evaluated for each of the three converted frequencies. (The *ϵ*_*zz*_ is the same as *ϵ*_*xx*_.) It is evident that the medium retains its anisotropic nature; however, for each of the frequencies, the nature of anisotropy is different. For *ω*_2,1_ and *ω*_2,2_, the components of the permittivity tensor exhibit opposite signs, indicating a hyperbolic nature of the medium. In contrast, at frequency *ω*_2,3_, all components of the permittivity tensor are positive (and less than unity), implying that the anisotropy at this frequency is of elliptic type. Additionally, at frequencies *ω*_2,1_ and *ω*_2,2_, permittivity tensors have different negative components, i.e., *ϵ*_*xx*,1_ and *ϵ*_*yy*,2_ are negative for *ω*_2,1_ and *ω*_2,2_, respectively, indicating that the isofrequency curves of the material for these two frequencies are rotated by *π*/2.

### 3.1 Band topology

From the previous, one can see that *ω*_2,2_(*ψ* = *π*/2) and *ω*_2,3_(*ψ* = 0) result from *pure* Drude and Lorentz dispersion, respectively, and thus *ω*_2,1_(*ψ* = 0) is the second solution pair expected from a Lorentzian response (as shown in [64], *ω*_2,3_ > *ω*_1_ can be connected to the resonant frequency *ω*_0,*yy*_ > *ω*_1_, whereas *ω*_2,1_ < *ω*_1_ approaches the solution in a nondispersive time interface [7]). On the other hand, *ω*_2,1_(*ψ* = *π*/2) = 0 gives us the missing “wiggler” mode from the Drude plasma. For arbitrary *ψ*, these three *pure* frequencies are hybridized into three different solutions, considering that sum of them squared is a conserved quantity independent of *ψ*, reduced when *ϵ*_∞_ = 1 to:

(8) $$\omega_{2,1}^{2}+\omega_{2,2}^{2}+\omega_{2,3}^{2}=\omega_{1}^{2}+\left(1+f\right)\omega_{\text{p}}^{2}.$$

**Figure 3:**
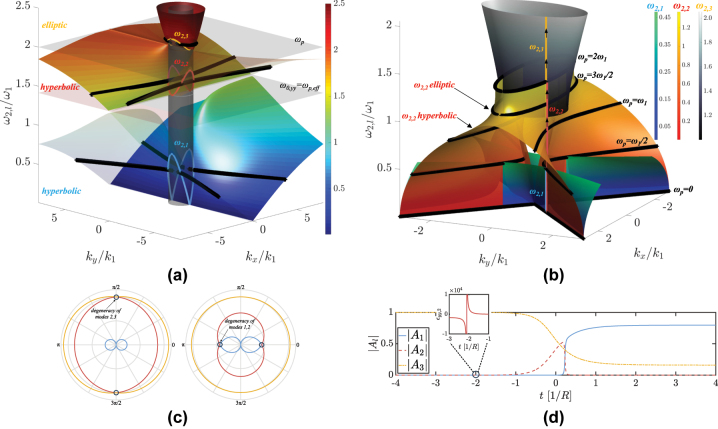
Dispersion topology of the homogenized hyperbolic material. (a) Dispersion surface of the three bands when *d*_*m*_ = *d*_*d*_: from momentum conservation, their intersection with *ω*_1_ light in vacuum (cylindrical surface) reveals the excited states after the temporal interface; corresponding isofrequency plots are indicated in black for *ψ* = *π*/4. (b) Continuum of isofrequency contours (*d*_*m*_ = *d*_*d*_/2) that correspond with the blueshifting evolution of the excited states *ω*_2,*l*_ as the plasma frequency goes from 0 to 2*ω*_1_. Each *ω*_2,*l*_ renders a different surface, with its own colormap. The black isofrequency contours indicate a transition from hyperbolicity to ellipticity within the second band (*ω*_2,2_). The zenithal view of the inset shows how the forbidden directions of the first and second bands are perpendicular. (c) Coalescence of excited states from bands 2 and 3 when *ψ* = *π*/2 (left), and from bands 1 and 2 when *ψ* = 0 (right). This happens when *f* = 1 − *κ* ^2^ (e.g., *d*_*m*_ = 3*d*_*d*_ for *ω*_p_ = 2*ω*_1_) and $f=\frac{1+2\kappa^{2}-\sqrt{1+4\kappa^{4}}}{2}$ , respectively, with *κ* = *ω*_1_/*ω*_p_. (d) Time evolution of the FW amplitudes when a perfectly abrupt step-function temporal interface is replaced by a deeply subcycle sigmoid function, with *ψ* = 1°: during this transition, the matched wave in band 2 (red) first feels a hyperbolic medium and grazes the corresponding forbidden direction, so it is barely coupled to. As *ω*_p_ keeps increasing, the medium becomes elliptic in the new excited portion of band 2 (out-of-plane dielectric tensor element shown in the inset), and the wave amplitude |*A*_2_| begins to increase before tunneling out to band 1 (this tunneling effect is explained in Section 5).

The dispersion diagram of the new homogenized material in Figure 3(a) reveals three distinct frequency bands. (i) A hyperbolic lower band in the range [0, min(*ω*_0,*yy*_, *ω*_p,eff_)]. (ii) A mid band in the range [*ω*_p,eff_, *ω*_p_], which is elliptic (hyperbolic) below (above) resonance *ω*_0,*yy*_. (This requires that *ω*_0,*yy*_ > *ω*_p,eff_, only satisfied if *d*_*m*_ < *d*_*d*_; otherwise, if *ω*_0,*yy*_ < *ω*_p,eff_, a bandgap opens between the first and the second bands. In Figure 3(a), *d*_*m*_ = *d*_*d*_, so this band is only hyperbolic and there is no gap underneath. Coincidentally, moreover, *ω*_0,*yy*_ = *ω*_p,eff_ equate the surface plasmon frequency $\omega_{\text{p}}/\sqrt{2}$ of the actual metallic layers.) (iii) An elliptic upper band above *ω*_p_. In this figure, the cylinder’s surface encompasses all the possible angular directions of the initial wave in vacuum, so its intersection with the three bands provides the necessary momentum matching condition (such three intersections (blue, red, and yellow lines) are precisely the curves in Figure 2(a)). Moreover, the intersection of the dispersion surface with the horizontal planes *ω* = *ω*_2,*l*_ (chosen here for *ψ* = *π*/4) yields the three corresponding isofrequency contours (black curves).

Instead of the material dispersion in its final state, Figure 3(b) superimposes the isofrequency contours that correspond to each of the *ω*_2,*l*_ eigenstates, as they increase with increasing plasma frequency (from 0 to its final value after the transition) for a fixed propagating angle (*ψ* = *π*/4). Each of the three surface contours *ω*_2,*l*_ is plotted with a different colormap and shows how the second band makes a transition from hyperbolic to elliptic in its excited frequency *ω*_2,2_ when *d*_*m*_ < *d*_*d*_ (we here choose *d*_*m*_ = *d*_*d*_/2). In the limit of *ω*_p_ → ∞, $\omega_{2,1}\to \left[0,\sqrt{1-f}\omega_{1}\right]$ depending on *ψ*, $\omega_{2,2}\to \sqrt{f}\omega_{\text{p}}$ , and *ω*_2,3_ → *ω*_p_, which satisfies Eq. (8).

Now that the interplay between *ω*_1_/*ω*_p_ and *f* has been visualized, further insight is gained by noticing that *ω*_2,2_(*ψ* = 0) = *ω*_p,eff_, i.e., at *ψ* = 0 this mode of zero field amplitude becomes the effective bulk plasmon resonance *ϵ*_*xx*,2_ = 0, which is thus not coupled onto. Likewise, *ω*_2,3_(*ψ* = *π*/2), of zero field amplitude also, becomes $\sqrt{\omega_{\text{p,eff}}^{2}+\omega_{0,yy}^{2}}$ (which is simply *ω*_p_ when *ϵ*_∞_ = 1) and, therefore, *ϵ*_*yy*,3_ = 0. However, the described behavior holds as long as *f* < 1 − *κ* ^2^ (below resonance, i.e., *ω*_1_ < *ω*_0,*yy*_) and $f>\frac{1+2\kappa^{2}-\sqrt{1+4\kappa^{4}}}{2}$ , *κ* being *ω*_1_/*ω*_p_. Right at the upper threshold for *f*, *ω*_2,2_ and *ω*_2,3_ become degenerate and equal to *ω*_p_ when *ψ* = *π*/2 (Figure 3(c), left panel). Above this threshold, the incident wave does couple to *ω*_2,3_ when *ψ* = *π*/2, but instead there is no coupling to *ω*_2,2_ when *ψ* is either 0 or *ψ* = *π*/2. In the limit *f* → 1, coupling to *ω*_2,2_ vanishes for all directions: the Lorentz mode *ω*_2,3_ plays the role of an effective isotropic plasma, since *ω*_0,*yy*_ → 0, and *ω*_2,1_ → 0 describes the associated DC mode for all *ψ*. Similarly, at the lower threshold, *ω*_2,1_ and *ω*_2,2_ coalesce into *ω*_p,eff_ (Figure 3(c), right panel). Below this threshold, when *ψ* = 0, there is no coupling to *ω*_2,1_ but there is to *ω*_2,2_. Taking the limit *f* → 0 (no temporal boundary at all), there is only FW coupling to *ω*_2,2_ → *ω*_1_, regardless of *ψ*, given that

(9) $$\omega_{2,1}\to 0,\;\omega_{2,2}\to \omega_{1},\;\omega_{2,3}\to 2\omega_{1}.$$

In short, the incoming wave feels vacuum at *ω*_2,2_ in the new medium parameterized by *ω*_p,eff_ → 0 and *ω*_0,*yy*_ → 2*ω*_1_, again assuming *ϵ*_∞_ = 1. This vacuum limit is different if, e.g., one recovers *d*_d_ = *d*_*m*_ and makes *ω*_p_ → 0, in which case

(10) $$\omega_{2,1}\to 0,\;\omega_{2,2}\to 0,\;\omega_{2,3}\to \omega_{1}.$$

In such scenario, the incoming wave couples to the new medium – where the resonance frequency *ω*_0,*yy*_ now also vanishes – through *ω*_2,3_. These limiting cases boil down to the vanishing width of the first and second bands as *f* → 0 and *f* → 1, respectively.

From Figure 3(a) and (b), propagation of the *ω*_2,2_ eigenmode is forbidden along the *x* direction in its hyperbolic phase. In order to see this elliptic-to-hyperbolic transition, we choose *d*_*m*_ = *d*_*d*_/2 and *ψ* = 1° and solve the temporal transition in the eigenstate basis versus time, assuming a subcycle sigmoid function of transition rate *R* for the parameterization of the time-varying *ω*_p_. As seen in Figure 3(d), only after the transition of *ϵ*_*yy*,2_ from positive to negative, at *t* ≈ − 2/*R* (see inset), does the FW mode amplitude |*A*_2_| (red dashed) begin to increase (a detailed derivation of the complex field amplitudes is found in the next section).

## 4 Field amplitudes after temporal interface

To get a better insight of the wave phenomena after the abrupt emergence of anisotropy, one needs to determine amplitudes of the fields after the temporal jump. The fields after an abrupt temporal interface consist of the three pairs of FW and BW waves. To determine their six amplitudes, we need to have six independent temporal boundary conditions. From our homogenized model of anisotropy in Eq. (2), the second-order nature of the time-varying Drude and Lorentzian response in the form of two separate second-order differential equations for the *x* and *y* components of the polarization density vector **P** and electric field vector **E** can be written as

(11a) $$\frac{dP_{x}^{2}\left(t\right)}{dt^{2}}=\epsilon_{0}\omega_{\text{p,eff}}^{2}\left(t\right)E_{x}\left(t\right),$$

(11b) $$\frac{dP_{y}^{2}\left(t\right)}{dt^{2}}+\omega_{0,yy}^{2}\left(t\right)P_{y}\left(t\right)=\epsilon_{0}\omega_{\text{p,eff}}^{2}\left(t\right)E_{y}\left(t\right),$$

where *ϵ*_∞_ is assumed to be unity. Therefore, temporal continuity of *P*_*x*_ and *P*_*y*_ and their time derivatives at the temporal interface is implied. Together with temporal continuity of electric and magnetic flux densities, **D** = *ϵ*_0_ **E** + **P** and **B** = *μ*_0_ **H**, we have a set of six temporal boundary conditions. Worth mentioning that continuity of *D*_*x*_ and *D*_*y*_ yields the same equations (as an aside, it is worth noting that the temporal continuity of **P** and **D** automatically guarantees the temporal continuity of **E**). Let us assume that before the temporal jump at *t* = *t*_0_, we have

(12a) $$H_{1}=ze^{j\omega_{1}t}e^{-jk\cdot r},$$

(12b) $$E_{1}=\frac{1}{\epsilon_{0}\omega_{1}\epsilon_{\text{r},1}}\left(-xk_{y}+yk_{x}\right)e^{j\omega_{1}t}e^{-jk\cdot r},$$

(12c) $$D_{1}=\frac{1}{\omega_{1}}\left(-xk_{y}+yk_{x}\right)e^{j\omega_{1}t}e^{-jk\cdot r},$$

(12d) $$\frac{dP_{1}}{dt}=j\frac{\epsilon_{\text{r},1}-1}{\epsilon_{\text{r},1}}\left(-xk_{y}+yk_{x}\right)e^{j\omega_{1}t}e^{-jk\cdot r},$$

where subscript “1” indicates the quantities before *t*_0_. For the sake of simplicity, let us assume *t*_0_ = 0, then quantities after *t*_0_ are denoted with subscript “2” and they can be written as

(13a) $$H_{2}=ze^{-jk\cdot r}\overset{3}{\underset{l=1}{\sum }}\left(A_{l}e^{j\omega_{2,l}t}-B_{l}e^{-j\omega_{2,l}t}\right),$$

(13b) $$\begin{array}{ll} E_{2} & =\frac{e^{-jk\cdot r}}{\epsilon_{0}}\overset{3}{\underset{l=1}{\sum }}\left(-x\frac{k_{y}}{\epsilon_{xx,l}}+y\frac{k_{x}}{\epsilon_{yy,l}}\right)\\ & \;\times \frac{1}{\omega_{2,l}}\left(A_{l}e^{j\omega_{2,l}t}+B_{l}e^{-j\omega_{2,l}t}\right), \end{array}$$

(13c) $$\begin{array}{ll} D_{2} & =e^{-jk\cdot r}\overset{3}{\underset{l=1}{\sum }}\left(-xk_{y}+yk_{x}\right)\\ & \;\times \frac{1}{\omega_{2,l}}\left(A_{l}e^{j\omega_{2,l}t}+B_{l}e^{-j\omega_{2,l}t}\right), \end{array}$$

(13d) $$\begin{array}{ll} \frac{dP_{2}}{dt} & =je^{-jk\cdot r}\sum_{l=1}^{3}\left(-x\frac{k_{y}\left(\epsilon_{xx,l}-1\right)}{\epsilon_{xx,l}}\right.+\left.y\frac{k_{x}\left(\epsilon_{yy,l}-1\right)}{\epsilon_{yy,l}}\right)\\ & \;\times \left(A_{l}e^{j\omega_{2,l}t}-B_{l}e^{-j\omega_{2,l}t}\right). \end{array}$$

Using equations in (12) and (13) and the six temporal boundary conditions, one can write the following expressions:

(14) $$\left(\begin{array}{cccccc} 1 & -1 & 1 & -1 & 1 & -1\\ \frac{1}{\omega_{2,1}} & \frac{1}{\omega_{2,1}} & \frac{1}{\omega_{2,2}} & \frac{1}{\omega_{2,2}} & \frac{1}{\omega_{2,3}} & \frac{1}{\omega_{2,3}}\\ \frac{1}{\omega_{2,1}\epsilon_{xx,1}} & \frac{1}{\omega_{2,1}\epsilon_{xx,1}} & \frac{1}{\omega_{2,2}\epsilon_{xx,2}} & \frac{1}{\omega_{2,2}\epsilon_{xx,2}} & \frac{1}{\omega_{2,3}\epsilon_{xx,3}} & \frac{1}{\omega_{2,3}\epsilon_{xx,3}}\\ \frac{1}{\omega_{2,1}\epsilon_{yy,1}} & \frac{1}{\omega_{2,1}\epsilon_{yy,1}} & \frac{1}{\omega_{2,2}\epsilon_{yy,2}} & \frac{1}{\omega_{2,2}\epsilon_{yy,2}} & \frac{1}{\omega_{2,3}\epsilon_{yy,3}} & \frac{1}{\omega_{2,3}\epsilon_{yy,3}}\\ \frac{\epsilon_{xx,1}-1}{\epsilon_{xx,1}} & -\frac{\epsilon_{xx,1}-1}{\epsilon_{xx,1}} & \frac{\epsilon_{xx,2}-1}{\epsilon_{xx,2}} & -\frac{\epsilon_{xx,2}-1}{\epsilon_{xx,2}} & \frac{\epsilon_{xx,3}-1}{\epsilon_{xx,3}} & -\frac{\epsilon_{xx,3}-1}{\epsilon_{xx,3}}\\ \frac{\epsilon_{yy,1}-1}{\epsilon_{yy,1}} & -\frac{\epsilon_{yy,1}-1}{\epsilon_{yy,1}} & \frac{\epsilon_{yy,2}-1}{\epsilon_{yy,2}} & -\frac{\epsilon_{yy,2}-1}{\epsilon_{yy,2}} & \frac{\epsilon_{yy,3}-1}{\epsilon_{yy,3}} & -\frac{\epsilon_{yy,3}-1}{\epsilon_{yy,3}} \end{array}\right)\cdot \left(\begin{array}{c} A_{1}\\ B_{1}\\ A_{2}\\ B_{2}\\ A_{3}\\ B_{3} \end{array}\right)=\left(\begin{array}{c} 1\\ \frac{1}{\omega_{1}}\\ \frac{1}{\omega_{1}\epsilon_{\text{r,}1}}\\ \frac{1}{\omega_{1}\epsilon_{\text{r,}1}}\\ \frac{\epsilon_{\text{r,}1}-1}{\epsilon_{\text{r,}1}}\\ \frac{\epsilon_{\text{r,}1}-1}{\epsilon_{\text{r,}1}} \end{array}\right).$$

**Figure 4:**
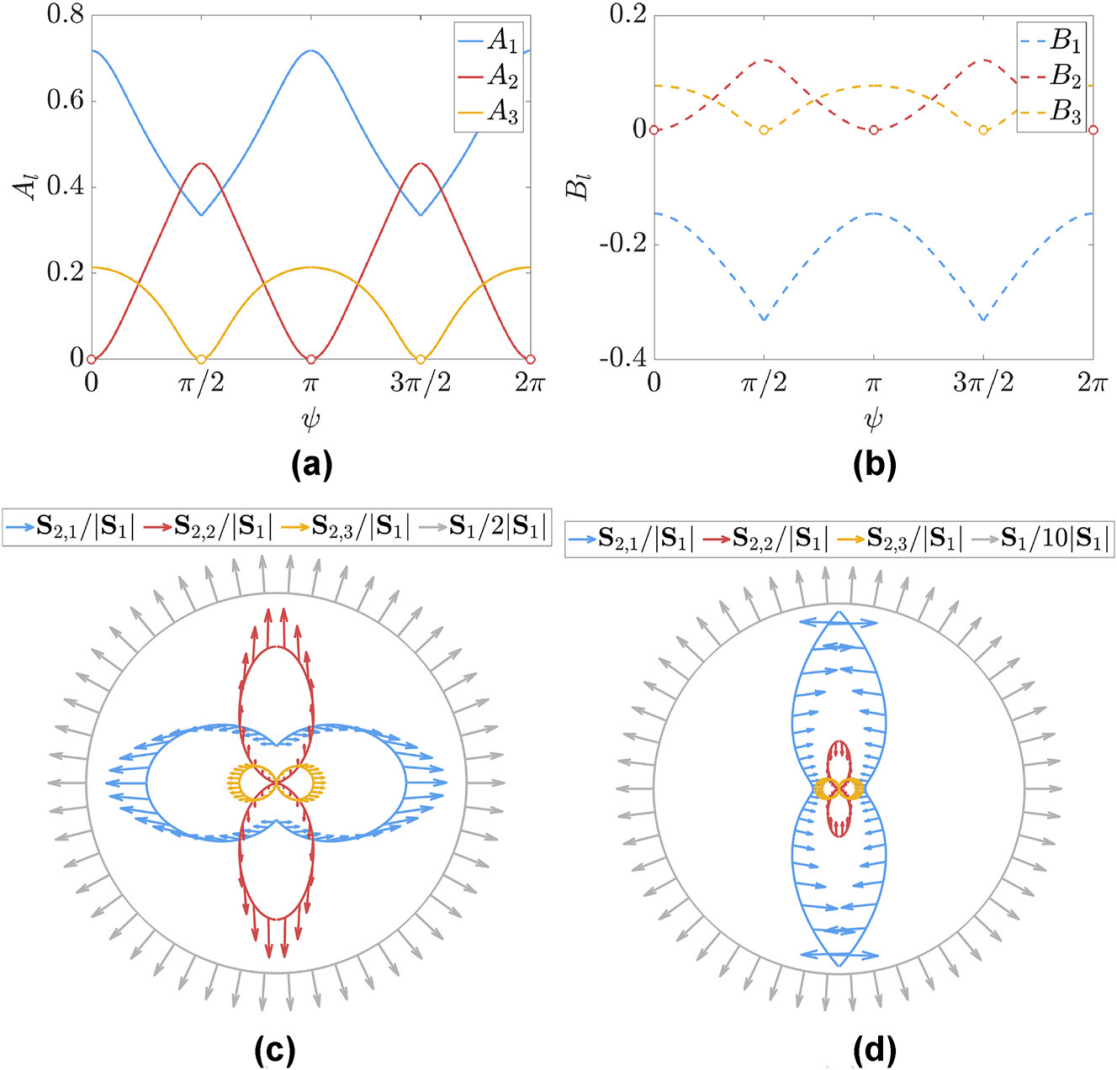
Field amplitudes and time-averaged Poynting vectors after the temporal interface: field amplitudes for (a) FW waves, *A*_*l*_, and (b) BW waves, *B*_*l*_; (c) time-averaged Poynting vector of FW for each converted frequency as well as the initial Poynting vector divided by 2; (d) time-averaged Poynting vector of BW for each converted frequency as well as the initial Poynting vector divided by 10. (Since the Poynting vectors for FW and BW waves are small compared to *S*_1_, in order to show their details, we scale down *S*_1_ in the figure.)

Here, each line of the matrix from top to bottom corresponds to continuity of *B*_*z*_, *D*_*x*_ (or *D*_*y*_, same equation), *E*_*x*_ (or *P*_*x*_), *E*_*y*_ (or *P*_*y*_), $\frac{dP_{x}}{dt}$ and $\frac{dP_{y}}{dt}$ , respectively. Solving this system of equations provides field amplitudes for FW and BW waves. Figure 4(a) and (b) shows linear plots of field amplitudes as a function of the direction of propagation angle *ψ*. Figure 4(c) and (d) shows the amplitude and direction of the time-averaged Poynting vector of FW and BW for each converted frequency. Directions of arrows indicate the direction of the Poynting vector. Colored contours and length of the arrows indicate the amplitude of the Poynting vector of the corresponding frequency branch.

From Figures 2 and 4, one can make several observations: First – for the limiting cases *ψ* = 0 and *ψ* = *π*/2 (and obviously also for *ψ* = *π* and *ψ* = 3*π*/2), only two sets of wave have nonzero amplitudes, since *A*_2_ = *B*_2_ = 0 at *ψ* = 0 (and *ψ* = *π*) and *A*_3_ = *B*_3_ = 0 at *ψ* = *π*/2 (and *ψ* = 3*π*/2). This results in zero time-averaged Poynting vector *S*_2,2_ for FW and BW waves for *ψ* = 0 (and *ψ* = *π*) and *S*_2,3_ for *ψ* = *π*/2 (and *ψ* = 3*π*/2). For all other angles of propagation, we always get three pairs of FW and BW waves. Second – the first set for *ω*_2,1_ is always nonzero, i.e., *A*_1_ ≠ 0 and *B*_1_ ≠ 0 for all *ψ*. Third – for the first converted frequency branch, while *ϵ*_*xx*,1_ becomes negative (and infinitely large for *ψ* = *π*/2 (and *ψ* = 3*π*/2)), the corresponding wave always exists and its converted frequency *ω*_2,1_ approaches zero for *ψ* = *π*/2 (and *ψ* = 3*π*/2), which essentially means DC field. However, we note that while the field amplitudes *A*_1_ and *B*_1_ at angle *ψ* = *π*/2 (and *ψ* = 3*π*/2) are not zero, the *x* component of the electric field is zero since the expression *ω*_2,1_ *ϵ*_*xx*,1_ in the denominator approaches infinitely large value as *ω*_2,1_ approaching zero at *ψ* = *π*/2 (and *ψ* = 3*π*/2) (see Eqs. (13a) and (13b)). Effectively for *ψ* = *π*/2 (and *ψ* = 3*π*/2), we have a Drude medium, where the first solution is a DC (effectively “frozen”) magnetic field (while the *xx* element of relative permittivity for this set is infinitely negative, thus behaving as a perfect electric conductor causing the *x* component of the electric field to be zero) and the second solution is a propagating wave. Fourth, it is worth noting that for waves propagating along *ψ* = *π*/2 (and *ψ* = 3*π*/2), the medium after the temporal jump behaves as a Drude medium (with *A*_1_ ≠ 0, *B*_1_ ≠ 0, *A*_2_ ≠ 0 and *B*_2_ ≠ 0), but for waves propagating along *ψ* = 0 (and *ψ* = *π*), the medium behaves as a Lorentzian medium (with *A*_1_ ≠ 0, *B*_1_ ≠ 0, *A*_3_ ≠ 0 and *B*_3_ ≠ 0). For all other angles, after the temporal jump, the medium behaves as a medium with a dispersion not resembling solely Drude or Lorentzian type, with all three pairs of amplitudes *A*_1_ ≠ 0, *B*_1_ ≠ 0, *A*_2_ ≠ 0, *B*_3_ ≠ 0, *A*_3_ ≠ 0, and *B*_3_ ≠ 0. Finally, the Poynting vector in Figure 4(c) and (d) demonstrates another interesting insight on the wave properties. One notices that for all three converted frequencies, the corresponding energy flows mainly along the optical axes, which can be attributed to a certain level of canalization of energy in this system. We point out that along the directions *ψ* = *π*/2 and *ψ* = 3*π*/2, the Poynting vector for the converted frequency *ω*_2,1_ (which is near zero) is identically zero (since the *x* component of the electric field is zero for this set). Therefore, the blue-color plots of the Poynting vector in Figure 4(c) and (d) have a zero value along those directions, but attain nonzero values when the direction of propagation deviates from *ψ* = *π*/2 and *ψ* = 3*π*/2 with its flow being primarily along the *x* axis.

## 5 Numerical simulation

**Figure 5:**
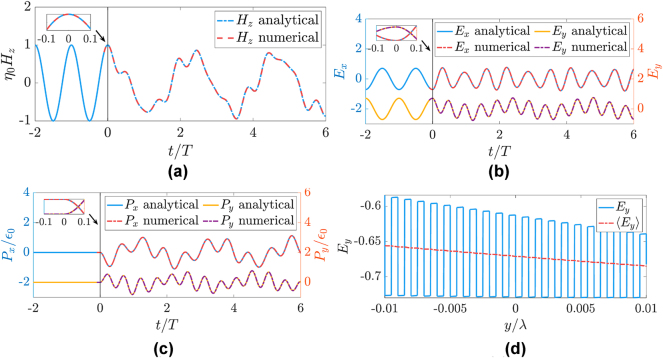
Comparison of analytical and numerical results. (a–c) Show magnetic field (*H*_*z*_), *x* and *y* components of electric field (*E*_*x*_ and *E*_*y*_), and *x* and *y* components for polarization density (*P*_*x*_ and *P*_*y*_) obtained analytically and numerically. Insets in (a–c) highlight continuity of the plotted quantities across the temporal interface. (d) Electric field *E*_*y*_ along *y* axis when *x* = 0 ( $e^{-jk_{x}x}$ dependence, as seen in Eq. (15)), which exhibits discontinuities, and its homogenized version ⟨*E*_*y*_⟩ (resulting from *y*-averaging over a distance of *λ*/100 or, equivalently, about 10 unit cells) agreeing with effective medium theory.

One may argue that, rigorously speaking, the continuity of the *homogenized* d**P**/d*t* adopted above might still be, at least in principle, open to debate for the following reasons: (i) it is true that, within each metal layer with the Drude dispersion, a temporal discontinuity in the plasma frequency leads to temporally continuous nonhomogenized d**P**/d*t* when the Drude current response follows; (ii) it is also clear that Eq. (11) correctly models the homogenized anisotropic response once *ω*_p_ (and thus *ω*_p,eff_ and *ω*_0,*yy*_) is time-invariant. But the assumption that these same equations are still indeed valid across the time interface, when *ω*_p_(*t*) varies in time, should be tested. To the best of our knowledge, a theory of time-varying homogenization in the presence of dispersion has not been done yet. If Eq. (11) were not to be *exact* (in the following we prove they are), different temporal boundary conditions might be applicable for the homogenized quantities. In light of this, in order to validate our analytic derivations, we numerically solve homogenized model described by Eq. (11) and the actual nonhomogenized problem with deeply subwavelength Drude layers. We present the results only for the latter case, since the results for the former case of homogenized model are identical with the analytical results, if the layers are thin enough (∼*λ*/10^3^ in our case). Regardless of the model, the in-plane momentum *k*_*x*_ allows for a dimensionality reduction, so only *y* needs to be parameterized. We can thus write

(15) $$H_{z}\left(x,y,t\right)={\tilde{H}}_{z}\left(y,t\right)e^{-jk_{x}x},$$

with phasor ${\tilde{H}}_{z}\left(y,t\right)=e^{j\omega_{1}t}e^{-jk_{y}y}$ before the temporal transition, in an unbounded vacuum. We can thus focus on the time evolution of the *k*-phasors, which abides by the curl equations

(16) $$\begin{array}{cc} & \left(\begin{array}{ccc} 0 & -\partial_{y} & -jk_{x}\\ \partial_{y} & 0 & 0\\ jk_{x} & 0 & 0 \end{array}\right)\left(\begin{array}{c} {\tilde{H}}_{z}\\ {\tilde{E}}_{x}\\ {\tilde{E}}_{y} \end{array}\right)\\ & \;=\left(\begin{array}{ccc} -\mu_{0} & 0 & 0\\ 0 & \epsilon_{0} & 0\\ 0 & 0 & \epsilon_{0} \end{array}\right)\partial_{t}\left(\begin{array}{c} {\tilde{H}}_{z}\\ {\tilde{E}}_{x}\\ {\tilde{E}}_{y} \end{array}\right)+\left(\begin{array}{c} 0\\ {\tilde{J}}_{x}\\ {\tilde{J}}_{y} \end{array}\right), \end{array}$$

and the nonhomogenized time-varying current response

(17) $$\partial_{t}{\tilde{J}}_{x/y}=\epsilon_{0}\omega_{\text{p}}^{2}\left(y,t\right){\tilde{E}}_{x/y},$$

where $\omega_{\text{p}}^{2}\left(y,t\right)$ is nonzero only inside the Drude layers, depending on *y*. Using finite differences in both *y* and *t* and a Yee grid, Eqs. (16) and (17) can be solved in leapfrog fashion [65]. Figure 5 shows the temporal evolution of the fields at *x* = *y* = 0 when *ψ* = *π*/4, with perfect overlap of the analytic and numerical curves, demonstrating that our assumption of temporal continuity used above for the homogenized quantities is indeed valid. An exceedingly small unit cell of *a* = *λ*/10^3^ (*y* step of *a*/200) is chosen in order to keep the spectral content within the long-wavelength portion of the first band in the dispersion diagram, such that the homogenization behind our analytic expressions is applicable. (Anyhow, regardless of *a*, we should point out that out-of-plane momentum *k*_*y*_ is partially conserved too, in that ${\tilde{H}}_{z}\left(y+a,t\right)=e^{-jk_{y}a}{\tilde{H}}_{z}\left(y,t\right)$ at all times. That is, the crystal momentum – i.e., the portion of *k*_*y*_ within the first Brillouin zone – is conserved, and any temporal variation of this crystal, as long as it is structured in *y* with *a* periodicity, will only contribute to the total out-of-plane momentum with integer multiples of the reciprocal lattice vector 2*π*/*a*. The solution could thus be expanded as a Bloch wave, or else, as in our case, one can simply simulate one unit cell in the finite-differences solver by imposing the Bloch boundary condition.) Accordingly, the numerical curves for *E*_*y*_ and *P*_*x*/*y*_, discontinuous across the successive air/metal interfaces, follow from *y*-averaging over a few unit cells. Figure 5(d) illustrates these discontinuities in *E*_*y*_(*t* = 6*T*) near *y* = 0.

**Figure 6:**
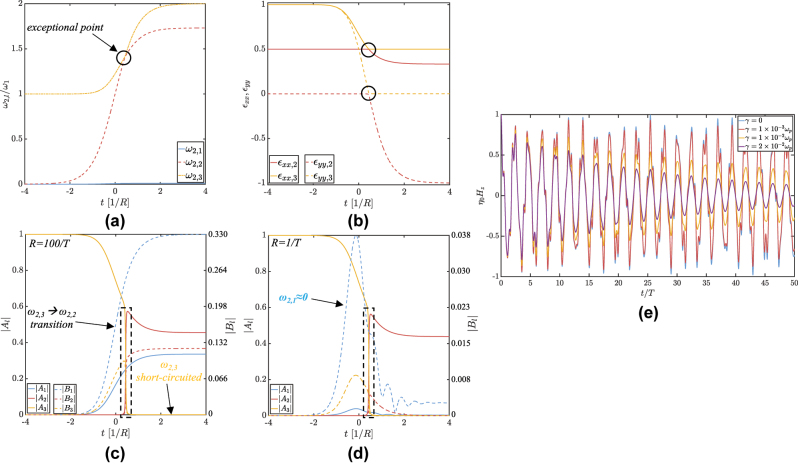
Impact of finite switching times and loss. (a) Evolution of the three eigenfrequencies as *ω*_p_ rises from 0 to 2*ω*_1_ versus time (normalized to the inverse of the sigmoid’s transition slope *R*), assuming *ψ* = 89° and *d*_*m*_ = *d*_*d*_. When *ω*_p_ satisfies $f=1-{\left(\omega_{1}/\omega_{\text{p}}\right)}^{2}$ , *ω*_2,2_ and *ω*_2,3_ become nearly degenerate (see black circle in the figure) and strongly interact: given that the initial mode *ω*_2,3_ in its final state is short-circuited by the metal–air interfaces, complete interband transition takes place from the third to the second level, as shown in panels (c) and (d). (b) Dielectric tensor elements of modes 2 and 3: at the degeneracy point, both out-of-plane permittivities *ϵ*_*yy*,2_ and *ϵ*_*yy*,3_ (dashed lines) become epsilon-near-zero (ENZ). (c) and (d) Temporal change of the FW (solid lines) and BW (dashed lines) field amplitudes for two different switching times: quasi-instantaneous (*R* = 100/*T*, panel (c)) and slow (*R* = 1/*T*, panel (d)). As *R* decreases, so does temporal reflection, with backward propagation tending to disappear. Still, the longer time of interaction between the two degenerate modes 2 and 3 favors frequency mixing down to mode 1 (DC), for which FW or BW waves lose their propagation character: we rather have a stationary pattern (dictated by the original wavenumber) that slowly oscillates in time. (e) Effect of adding some loss to the Drude model of the metallic layers: the evolution of the magnetic field phasor (*x* = *y* = 0) for several values of the nonradiative electron collision rate *γ* shows, over the first periods, very similar results to the ideal lossless material discussed so far. The decaying character of the (now complex) frequencies *ω*_2,*l*_ is nonetheless very clear after a few periods in the lossiest case, for which the actual metallic layer has a dielectric function of roughly −0.02*i* at the ENZ condition.

Finally, the effects of the finite switching time and loss have been studied and reported in Figure 6. Panels (c) and (d) illustrate how, as we reduce the rate of change *R* of the plasma frequency, which follows an arbitrarily fast (though continuous) sigmoid – particularly, the results in panel (c) are already very close to the previous analytic results for a perfectly abrupt time boundary – both temporal reflection (BW terms) and frequency splitting tend to decrease, as expected from the adiabatic limit picture. In contrast, evidently, the allowed frequencies across the transition (panel (a)), and therefore their associated permittivity tensors (panel (b)) are only function of *ω*_p_ and not of the rate *R*. Panel (e) depicts the temporal decay of the waves, following the imaginary part of the eigenfrequencies, when the Drude model of the metallic layers in Eq. (2) is extended with a damping term *γ*.

## 6 Conclusions

In summary, we have extended the notion of temporal interfaces to hyperbolic frequency dispersive media. Particularly, our analysis focused on a temporal interface between vacuum and a layered structure that exhibits hyperbolic properties. The interplay between anisotropy and frequency dispersion in this system was analyzed, which, together with a temporal interface, resulted in the splitting of the original wave into three different frequency pairs. We discussed the topological features in the dispersion of the effective time-varying anisotropic medium and its final properties at the new (converted) frequencies and noticed that it leads to energy canalization, i.e., propagation of power primarily along optical axes of the structure. Finally, full-wave simulations were conducted to corroborate our theoretical findings.

On the experimental side, several implementation scenarios can be envisioned. One possibility involves a periodic arrangement of dielectric and semiconductor layers, optically pumped to induce a temporal interface. The dielectric and semiconductor materials must be carefully selected so that the optical pumping leaves one medium unaffected while reconfiguring the other. As an alternative to the optical approach, one could consider a low-frequency, two-dimensional geometry – such as a metasurface. For instance, a hyperbolic metasurface operating at radio frequencies could be realized using lumped elements and switches, providing a feasible platform for proof-of-concept experiments.
